# Community perceptions on malaria and care-seeking practices in endemic Indian settings: policy implications for the malaria control programme

**DOI:** 10.1186/1475-2875-12-39

**Published:** 2013-01-29

**Authors:** Ashis Das, RK Das Gupta, Jed Friedman, Madan M Pradhan, Charu C Mohapatra, Debakanta Sandhibigraha

**Affiliations:** 1 The World Bank, Washington, DC, USA; 2 National Vector Borne Disease Control Programme, Ministry of Health and Family Welfare, Government of India, New Delhi, India; 3 Department of Health and Family Welfare, Government of Odisha, Bhubaneswar, India

**Keywords:** Malaria, Prevention, Treatment, Sociocultural belief, Community response, India

## Abstract

**Background:**

The focus of India’s National Malaria Programme witnessed a paradigm shift recently from health facility to community-based approaches. The current thrust is on diagnosing and treating malaria by community health workers and prevention through free provision of long-lasting insecticidal nets. However, appropriate community awareness and practice are inevitable for the effectiveness of such efforts. In this context, the study assessed community perceptions and practice on malaria and similar febrile illnesses. This evidence base is intended to direct the roll-out of the new strategies and improve community acceptance and utilization of services.

**Methods:**

A qualitative study involving 26 focus group discussions and 40 key informant interviews was conducted in two districts of Odisha State in India. The key points of discussion were centred on community perceptions and practice regarding malaria prevention and treatment. Thematic analysis of data was performed.

**Results:**

The 272 respondents consisted of 50% females, three-quarter scheduled tribe community and 30% students. A half of them were literates. Malaria was reported to be the most common disease in their settings with multiple modes of transmission by the FGD participants. Adoption of prevention methods was seasonal with perceived mosquito density. The reported use of bed nets was low and the utilization was determined by seasonality, affordability, intoxication and alternate uses of nets. Although respondents were aware of malaria-related symptoms, care-seeking from traditional healers and unqualified providers was prevalent. The respondents expressed lack of trust in the community health workers due to frequent drug stock-outs. The major determinants of health care seeking were socio-cultural beliefs, age, gender, faith in the service provider, proximity, poverty, and perceived effectiveness of available services.

**Conclusion:**

Apart from the socio-cultural and behavioural factors, the availability of acceptable care can modulate the community perceptions and practices on malaria management. The current community awareness on symptoms of malaria and prevention is fair, yet the prevention and treatment practices are not optimal. Promoting active community involvement and ownership in malaria control and management through strengthening community based organizations would be relevant. Further, timely availability of drugs and commodities at the community level can improve their confidence in the public health system.

## Background

Malaria is still a major global public health concern, despite many countries especially in the endemic Afro-Asian settings, having paid a considerable focus on its control [1]. India reports the highest malaria burden in the Southeast Asia region with 61% of the regional malaria cases [2]. In India, the malaria endemic central, eastern and north-eastern regions are characterized by substantial indigenous population, difficult terrains, low socio-economic development and less developed infrastructure [3,4].

Strengthening the availability of effective and affordable care has been a key strategy of all malaria-endemic countries [1]. However, these supply-side strategies were sub-optimally effective, as there was not adequate synergy between the service delivery and the community responses to it [1]. Thus, a concept has emerged as ‘community-based management of malaria’ with a thrust of positively shifting the community responses towards improvements in healthcare delivery [5]. Yet, as per the existing global evidence, such community approaches are ineffective to improve people’s care seeking, if their perceptions are not formulated and altered positively [6]. Among the known attributes of community perceptions and practices on malaria are their sociocultural and behavioural factors [6]. There is evidence that the availability of services alone may not ensure healthy practices, as they could be influenced by sociocultural barriers and inappropriate understanding of the disease aetiology [6]. Community perceptions and attitudes are essential inputs into healthy behaviours as they influence the pathways on symptom recognition, perceived disease seriousness, utilization of services, and eventual health outcomes [7]. In the context of a community-based approach, the understanding of community perceptions and practices are crucial for the policy makers to embed the disease control interventions into the socio-cultural dimensions of the community for effective adoption of healthy practices.

### Rationale

India has witnessed a slow reduction in disease burden, particularly of *falciparum* malaria, despite considerable investments on malaria control [5]. Recently, its malaria programme, embedded under the National Vector Borne Disease Control Programme (NVBDCP), has introduced a shift towards community-level management of malaria. Now, the village-based community health worker, known as accredited social health activist (ASHA), undertakes diagnosis and management of uncomplicated malaria in high-burden districts [8]. In addition, malaria prevention is supported by the introduction of long-lasting insecticide-treated nets (LLIN) [8]. However, little is known about the knowledge, attitude and practice on malaria and the determinants on community-based approaches in India. Further, qualitative studies providing deeper understanding of the pathways of health care seeking on malaria are scarce in the country.

This study aimed at generating evidence on the existing community perceptions, practices and their determinants on malaria control and management to complement the ongoing community-based malaria control programme. The study findings will help the programme for evidence-based policy development and programme management for effective malaria control. This qualitative exploration was undertaken in the State of Odisha. In 2010, Odisha contributed the highest number of malaria cases and deaths in India [3].

## Methods

### Study setting

**Figure 1:**
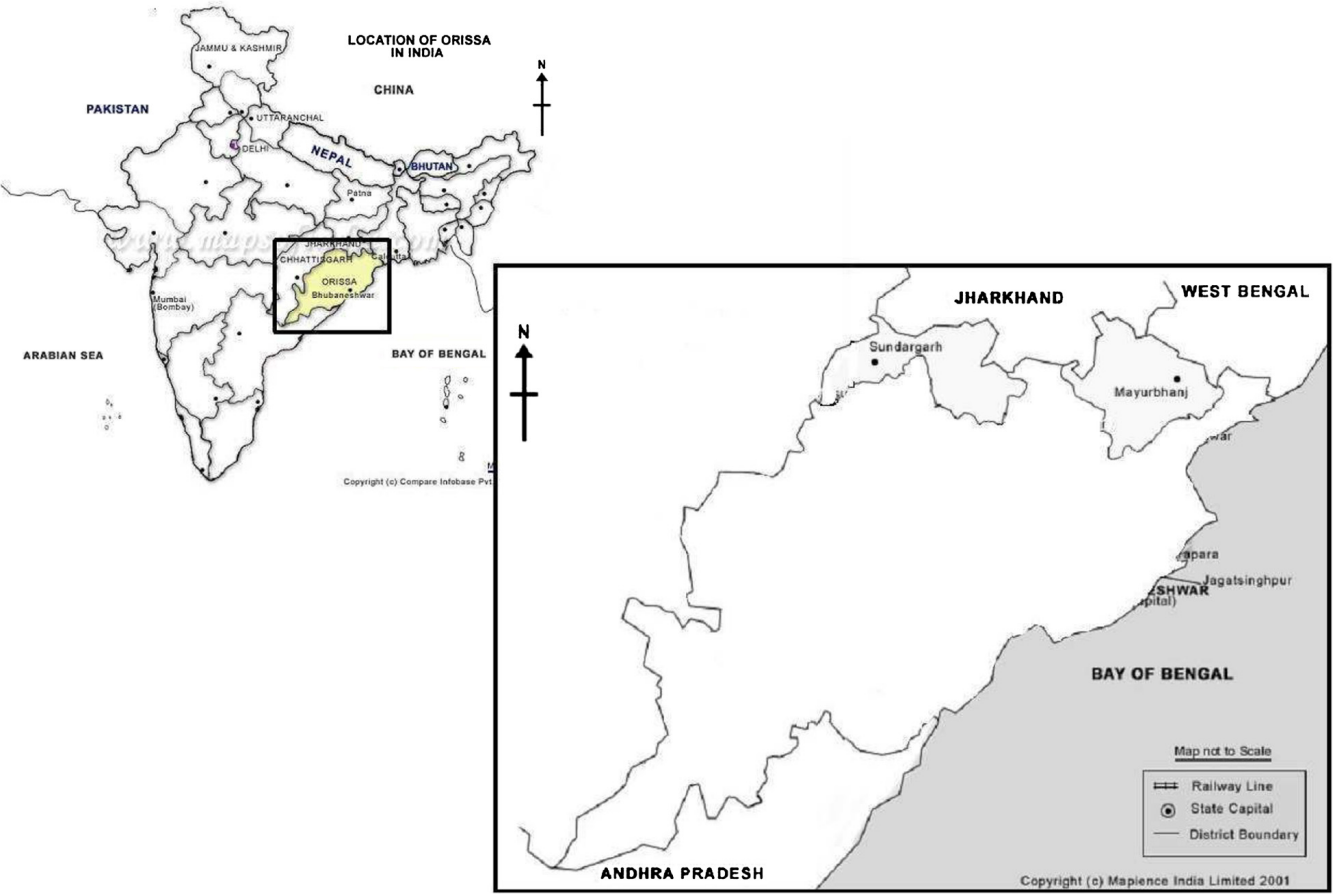
**Location of study area (Mayurbhanj and Sundargarh districts in Odisha State).** Source: http://www.mapsofindia.org.

The study was conducted between November 2009 and January 2010 in the districts of Mayurbhanj and Sundargarh of Odisha State (Figure 1). The NVBDCP selected these malaria-endemic districts to pilot the new programme interventions on community-based management of malaria through the ASHA. The north-eastern district Mayurbhanj is the largest district (area: 10,418 sq km) and has the third highest share of population (2,223,456) in the State [9]. It has a 51.9% literacy rate, 57% indigenous tribes, 42% forest cover and 7% urbanization [9]. The north-western district Sundargarh is the second largest district (area: 9,712 sq km) with the sixth highest population (1,830,673) in the State. It has 64.9% literacy rate, 50.2% indigenous tribes, 34% urbanization and 43% forest cover [10]. Subsistence farming and manual labour are the major economic activities of the inhabitants in these districts.

During 2009, Mayurbhanj and Sundargarh witnessed 10,798 and 20,796 malaria cases with eight and 12 reported deaths due to malaria respectively. More than 90% were falciparum malaria cases in both districts. Among the 30 Odisha districts, Sundargarh ranked seventh and Mayurbhanj was 15^th^ on the number of reported malaria cases in 2009 (State Malaria Office, Odisha, pers comm).

Each district is further administratively divided into ‘blocks’ with an average population of 100,000. Two such endemic blocks with annual parasite incidence above five (laboratory-confirmed malaria cases per 1,000 population) from each district were randomly selected for this study.

### Study design and sampling

This exploratory qualitative study was cross-sectional and employed focus group discussions (FGD) and key informant interviews (KII). A total of 26 FGDs and 40 KIIs were conducted in four endemic blocks in two districts. The number of interviews was decided on the basis of data saturation. There were separate FGD samples for adult men (Mayurbhanj n=4; Sundargarh n=5), adult women (Mayurbhanj n=5; Sundargarh n=4) and children aged 12 to 15 years (Mayurbhanj n=4; Sundargarh n=4), considering the cultural norm and opportunity for free expression of opinion. The discussions were organized at a common place such as community centres, schools, and community-based organizations (CBO) accessible to all socio-economic groups. The key informants were purposively selected according to their roles and responsibility with malaria service delivery and influence on the community in the study area. The key informant sample included district malaria officers (n=2), staff from non-government organizations (n=3), block medical officers (n=3), malaria laboratory technicians (n=3), female health workers (n=4), community health volunteers or ASHA (n=8), school teachers (n=4), traditional healers (n=4), less qualified providers (n=3), and local self-government functionaries (n=6).

### Data collection and analysis

The interview guides which were pre-tested on its content and duration guided the discussions. The discussions revolved around the themes on community perceptions, knowledge, practices regarding malaria prevention and treatment and factors affecting their perceptions and practices. The objective of KIIs was to understand the community’s perceptions and practices from the perspective of the service providers and opinion leaders. The first author conducted the FGDs and KIIs with the support of a local anthropologist researcher. The language of the discussions was the local language *Odia*. The FGDs took about 45 to 75 min and had nine to 12 participants each, whereas KIIs ran for 25 to 60 min. The participants were provided with light refreshments.

The FGDs and KIIs were digitally recorded. The recordings were transcribed and translated to English by two independent research assistants. The transcripts were later matched and merged to Microsoft Word. The electronic multimedia were transcribed within a week of interview and the initial transcripts further guided the researchers to modify the data collection tools. Each transcript was coded as per the coding matrix (deductive method) developed during the pretesting of the discussion guides. These codes along with additional new codes were organized (inductive method) according to various themes. The codes and the themes helped in arranging the views and opinions in a uniform manner. The transcribed data were subjected to content analysis [11]. Qualitative data analysis was performed with NVivo 8 software (QSR International Pty Ltd, Australia).

### Ethical considerations

The community members were informed about the aim of this research a week prior to the interviews either by their community leader, health volunteer or teacher. The key informants were contacted individually. Prior to each discussion or interview the purpose of the study and intended utilization of the information were explained to the participants. Risks and benefits of the study were explained and written informed consent (thumb imprints for non-literate participants) was obtained. Participation was voluntary and participants had the liberty to deny answering any question or withdraw at any point of time. All identities of the participants were removed during transcription and only opinions were presented. The study was conceived, planned and implemented in collaboration with the NVBDCP officials of the Department of Health and Family Welfare (DoHFW). Necessary approval was obtained from the DoHFW.

## Results

### Socio-demographic characteristics

The socio-demographic characteristics of the FGD participants are presented in Table 1. A total of 272 respondents with an equal gender representation were interviewed. Three-quarters of the sample belonged to the scheduled tribe community and about a half had some years of schooling. In terms of occupation, students were the majority (30%) followed by daily wage labourers (22.4%), farmers (21.3%) and homemakers (18.8%).

**Table 1:** Socio-demographic characteristics of the focus group discussion participants

**Variable**	**Mayurbhanj (%) (n=135)**	**Sundargarh (%) (n=137)**	**Total (%) (n=272)**
**Sex**			
Men	70 (51.9)	66 (48.2)	136 (50)
Women	65 (48.1)	71 (51.8)	136 (50)
**Age (years)**			
12-15	42 (31.1)	41 (29.9)	83 (30.5)
16-30	36 (26.7)	29 (21.2)	65 (23.9)
31-45	41 (30.4)	50 (36.5)	91 (33.5)
> 45	16 (11.9)	17 (12.4)	33 (12.1)
**Community**			
Scheduled caste ^a^	28 (20.7)	19 (13.9)	47 (17.3)
Scheduled tribe ^b^	95 (70.4)	108 (78.8)	203 (74.6)
Others	12 (08.9)	10 (07.3)	22 (08.1)
**Education (Years of schooling)**			
Non-literate (0)	48 (35.6)	73 (53.3)	121 (44.5)
Primary school (1–5)	59 (43.7)	43 (31.4)	102 (37.5)
High school and above (>6)	28 (20.7)	21 (15.3)	49 (18.0)
**Occupation**			
Farmer	32 (23.7)	26 (19.0)	58 (21.3)
Trader	4 (03.0)	5 (03.6)	9 (03.3)
Daily-wage labourer	21 (15.6)	40 (29.2)	61 (22.4)
Homemaker	28 (20.7)	23 (16.8)	51 (18.8)
Student	44 (32.6)	38 (27.7)	82 (30.1)
Not working	6 (04.4)	5 (03.6)	11 (04.0)

^a^socio-economically marginalized community, given special focus and privileges by the Government of India.

^b^socio-economically marginalized indigenous tribal population, given special focus and privileges by the Government of India.

### Local terminologies and illness perceptions

Malaria was locally known as ‘*meleria*’, a term derived from the biomedical nomenclature and there was no vernacular name. ‘*Meleria*’ included a cluster of symptoms closely resembling the biomedical presentation of malaria. All respondents ranked malaria as the most common disease or health condition in their locality. It was further reinforced by the healthcare providers and other key informants. Other perceived common ailments were diarrhoea, common cold, skin diseases, typhoid, and tuberculosis.

"Nowadays wherever you go, you would see ‘meleria’ patients. Whatever fever a person suffers from, the doctor tells it is ‘meleria’. [Male FGD participant, Sundargarh]"

"Malaria is the common illness in this area [Block medical officer, Mayurbhanj]"

The participants reported multiple causes of malaria. As shown in Table 2, although there were diverse responses, two represented the majority, i.e., dirty (contaminated) water and mosquitoes. Consuming unboiled or unfiltered water is thought to cause malaria. People who venture into the forest to collect firewood and forest produce are perceived to contract malaria through bathing and drinking water from forest rivulets. Participants who reported mosquitoes to be the cause had differences of opinions on how the mosquitoes spread the disease. Many opined that malaria was transmitted through mosquito bites. For some it was through exposure to food and water contaminated with infected mosquito eggs.

**Table 2:** Reported causes of malaria by the focus group discussion participants

**Perceived causes**	**Perceived transmission mechanism**	**Frequency (# FGDs mentioning out of total 24) N (%)**
1. contaminated water	A. Drinking	22 (91.7)
	B. Bathing in forest rivulets	14 (58.3)
	C. Drinking water from open well without boiling	15 (62.5)
2. Mosquitoes	A. Sucking blood	16 (66.7)
	B. Sitting on food and water	6 (25)
	C. Laying eggs on food and water	3 (12.5)
3. Environmental and personal sanitation and hygiene	Garbage	12 (50)
4. Stale food	Eating	11 (45.8)
5. Fatigue	Hard physical work and lack of rest	10 (41.7)
6. Housefly	Brings germs from garbage to food	9 (37.5)
7. Eating habit	Untimely eating	6 (25)
8. Untreated common cold	Unexplained	5 (20.8)
9. Change of season	Unexplained	4 (16.7)
10. Mother to baby	Unexplained	2 (8.3)
11. Blood	Transfusion of infected blood	1 (4.2)

"When we go to the forest, we have to drink water and take bath in the streams and rivulets. Upon our return we develop ‘meleria’. [Male FGD participant, Mayurbhanj]"

"Villagers do not cover the food items. When mosquitoes and flies sit on it, they contaminate the food. If one eats that food, it causes ‘meleria’. [Female FGD participant, Mayurbhanj]"

As discerned through the KII, health-care providers were aware of community perceptions and attributed the misconceptions regarding disease transmission to their low level of literacy and superstitions. A few informants were sceptical of the effectiveness of the current behaviour-change campaigns on community behaviour.

"You see…people here are illiterate and superstitious. Their level of awareness is very low. They have their own ideas for the aetiology of every disease, for instance, they say drinking contaminated water leads to malaria. [Medical Officer, Sundargarh]"

"We have been conducting so many awareness sessions in the community. Despite that we don’t see much improvement. [Malaria laboratory technician, Sundargarh]"

The FGD participants reported a higher incidence of malaria during the rainy season and the least during the dry period. Some could relate rains leading to more mosquito breeding sites and hence more malaria.

"In the rainy season we cultivate paddy. Water accumulates in the farms and we have plenty of mosquitoes. More mosquitoes mean more ‘meleria’. [Male FGD participant, Mayurbhanj]"

*‘Meleria’* in the locality was characterized by a combination of symptoms, closely resembling the clinical presentation of malaria. The FGD participants identified malaria as a febrile illness associated with severe shivering and headache (Table 3). All participants were able to state the symptoms. The majority perceived feeling cold, shivering, fever, intermittent fever, vomiting, and headache as malaria symptoms. Vomiting as a symptom was reported to be more commonly associated with childhood malaria. The female participants reported more malaria-specific symptoms than the men and the children. Participants were able to differentiate other fevers from malaria by the absence of its periodicity and shivering. Almost all participants reported the treatment by a physician at the primary health centre to be the more effective than any community-based provider.

**Table 3:** Reported symptoms of malaria by the focus group discussion participants

**Symptom**		**Women (n=8)**	**Men (n=8)**	**Children (n=8)**
**Local terminology (Odia language)**	**Literal English translation**			
*Thanda lagiba*	Feeling cold	8	8	8
*Deha thariba*	Shivering	8	5	4
*Banti haba*	Vomiting	8	2	6
*Deha batha*	Body ache	8	6	1
*Munda batha*	Headache	8	3	4
*Jara*	Fever	6	6	5
*Pali jara*	Intermittent fever	6	5	3
*Munda bulei haba*	Dizziness	2	2	1
*Durbala lagiba*	Weakness	2	2	0
*Bhoka na heba*	Loss of appetite	1	2	1
*Patala jhada*	Diarrhoea	0	0	4
*Kasa*	Cough	0	0	1
*Nakaru pani bohiba*	Running nose	0	1	0

"In ‘meleria,’ when the temperature goes up, the patient shivers, head becomes heavy and aches, whole body aches and vomiting takes place with the loss of appetite. The fever comes and goes on alternate days. [Female FGD participant, Sundargarh]"

Most respondents opined that malaria, if not treated timely will lead to jaundice, typhoid, *brain meleria* (cerebral malaria) and eventually death. The reported timeframe for developing these complications varied from six to seven days for typhoid, and to 12 to 14 days for jaundice and cerebral malaria.

"If a ‘meleria’ patient does not take medicines, the fever climbs up to the head and he behaves like mad. This is brain ‘meleria’, my father has told. [Female school student, Mayurbhanj]"

### Reported prevention modalities

Malaria prevention methods were reported to revolve around maintaining personal and environmental hygiene and drinking safe water.

"To prevent ‘meleria’, clothes should be clean, water should be covered and hands should be clean. [Female FGD participant, Sundargarh]"

"If we drink boiled water then we will not suffer from ‘meleria’. [Female school student, Sundargarh]"

The communitymembers perceived mosquitoes as a nuisance. All of them were reported to adopt some method of protection from mosquitoes during the rainy season when the vector is more prevalent. Among these methods, fumigating the house in the evenings with dried leaves, husk, straw, or firewood was reported to be the most common way of avoiding mosquitoes. Other reported prevention modalities were application of repellent oils out of neem (*Azadirachta indica*) and karanja (*Pongammia glabra*) seeds and burning anti-mosquito coils.

"We burn neem leaves and bark, cow dung cakes, dried leaves, grain husk to smoke away mosquitoes when they are too much. [Female FGD participant, Sundargarh]"

"We fumigate the house before we go to bed. Who cares after you are asleep? [Male FGD participant, Mayurbhanj]"

Though most were aware that mosquito nets can prevent malaria, only a few respondents used them regularly. The reported reasons for irregular use were the lack of adequate nets in the household due to unaffordablity, old or torn nets, a feeling of suffocation or heat inside the nets, exhaustion or intoxication at night that prevents proper use, and a preference to use nets for something else. FGDs respondents reported about using bed nets for fishing, filtering rice beer, setting traps to catch edible insects, and collecting sal leaves (*Shorea robusta*) to stitch leaf plates.

"Mosquito nets keep the mosquitoes away when we sleep and hence “meleria”. But, one big net (double size) costs 200 rupees (US$ 4) and we need many nets for a house as we are too many. From where shall we get this much money? [Male FGD participant, Mayurbhanj]"

"Alcoholism is a major problem in this region, which is an additional burden on the poverty. Here both men and women drink, though men more. They would borrow to drink than buying a mosquito net. When they are drunk they forget to hang the net at home, even they lie down on the road if they are too much drunk. [NGO staff, Mayurbhanj]"

"Mosquito nets have been given to them and they are not using it by telling it is too hot inside. Some even catch fish from the canals during the rains. [Female health worker, Mayurbhanj]"

If nets are few in a household, there is a preference for the children (at times with their mothers) to sleep under it. The reported use of bed nets was higher among children and women than men. There was no difference observed between the participants in both districts. The possessed nets were reported to be either never treated with an insecticide or treated at least a year ago. Around half of the participants were sceptical about the efficacy of nets to prevent malaria as they perceived mosquitoes were not the only cause and mosquitoes also bite during the non-sleeping hours. During the summer season, reported net use was less as it was hot and humid inside the nets. Most of the adult men slept out in the open, where it was difficult to hang the nets.

"What kind of protection do these nets give? Even with the nets hung, mosquitoes enter through the holes or suck blood from outside. When I wake up in the morning I see a lot of mosquitoes in my net with their bellies full of blood. Despite sleeping under the nets, my two children got ‘brain meleria’ six months back. [Male FGD participant, Mayurbhanj]"

### Reported care seeking for febrile illnesses

Despite developing fever and malaria-like symptoms, the majority of adult participants reported that care is not immediately sought for themselves. Rather they wait for a few days and engage in home remedies like consuming bitter herbal concoctions or a paste made from neem leaves. If the situation worsens they seek care from the local traditional healer.

"If we feel feverish, we think it might be weakness due to hard work. We wait and watch for two to three days. [Male FGD participant, Mayurbhanj]"

"Immediately they don’t come to me; suppose fever comes today then they won’t come today. If it continues further, they come to me after a couple of days. [ASHA, Sundargarh]"

The village-based traditional healers are not full-time professional health-care providers and most of them inherit the skills from their forefathers. In the locality, there were two types of traditional healers: *‘gunia’* (faith healer) and *‘baidya’* (herbalist). A *‘gunia’* resorted to sorcery and ritual blowing to ward off evil spirits. The *‘baidya’* on the other hand, cured ailments using roots, tubers, leaves and their concoctions. Some traditional healers used both principles. Care seeking from these healers is more of a reflection of faith and some even rely on them while simultaneously seeking care from other providers.

"First they go to ‘gunia’, perform ‘jhada-phunka’ (ritual blowing) and come to me after five to seven days. [Less qualified provider, Sundargarh]"

"People consume tablets and visit the ‘gunia’ at the same time; despite knowing that the tablet works. They have a faith that they should be treated by him (faith healer) at any cost. [ASHA, Mayurbhanj]"

Afterwards, depending upon the progression of disease and perceived severity, care is sought from other health care providers or facility, such as the community health worker, multipurpose village grocery shops stocking antipyretics (paracetamol), analgesics, and anti-malarials (chloroquine); less qualified provider (locally known as ‘private doctor’), and very rarely the primary health centre.

Care seeking for women and elderly, in general, was reported to be delayed. However, immediate care is sought for infants and children from the public health centres as there is a perceived notion of seriousness of their situation and inability of the local providers’ methods to ensure complete cure.

"Children are more vulnerable to malaria. We take our children immediately to the health centre when they get fever. ‘Private Doctors’ don’t have good medicines for the children; we can’t take risk by treating children at home through them. [Female FGD participant, Mayurbhanj]"

Care seeking from the less qualified providers (LQP) is very common considering their geographic vicinity, use of modern medicine and flexibility in modes of payment. Most LQPs are unqualified (without any education or training in medicine or allied health sciences), or less qualified (some education or training in allied health science), but legally are not allowed to practise modern medicine. Though the participants expressed their dissatisfaction with the providers’ attitude and cost of care, their choice of a more convenient alternative was limited. The majority of the participants felt the LQPs are overprescribing medicines for their own profit without considering the villagers’ financial hardship.

"With whatever fever we go to the ‘private doctor’, he tells it is ‘meleria’ and you have to take high potency injections. We don’t know much about the disease, so we have to obey him. [Male FGD participant, Mayurbhanj]"

The LQPs almost uniformly narrated the treatment for fever and malaria-like illness with an anti-malarial injection (arteether), an antibiotic, paracetamol, iron and multivitamin syrups. There is a perceived advantage of injections in the community as they think more pain during the treatment will give them a more effective cure. Also, the community perceives that the injection directly delivers the medicine in their blood stream, so it will give them quick relief and they will be able to resume their work early. On the other hand, the oral formulations would reach the blood through the stomach and some have prior experience of side effects like dizziness, vomiting, or tinnitus with tablets. That is why, in certain cases, the patients demand injections.

"Villagers believe that the more they have to undergo pain during treatment, the more effective it is. Though the tablets are cheaper; still the people are prepared to pay more for the injections. [Less qualified provider, Mayurbhanj]"

"With one injection it needs a day to recover as it goes directly to my blood, but consuming tablets will take at least two to three days. How my family will eat if I don’t go to work for those days? We don’t want to get into more trouble (drug side effects) by consuming tablets. [Male FGD participant, Sundargarh]"

The treatment for an episode of fever in this fashion costs around INR 300 to 700 (US$ 7–15), and in case of complicated malaria it can reach up to INR 2,000 to 3,000 (US$ 45–65). This level of health-care expenditure can severely burden an average rural family with one breadwinner engaged in subsistence farming or wage labour. The peak malaria transmission season (June to September) coincides with the “lean” period when income is at a seasonal low. At times households have to borrow from a moneylender with high interest rates or sell scarce assets such as land, jewellery, or livestock to arrange for the treatment. The growing presence of microfinance–related, women’s self-help groups have helped to alleviate this burden, but not reduced the cost of expenditure.

"If a card (rapid diagnostic test) test is done, followed by three injections of EMAL (arteether) and an antibiotic, the cost comes to Rs.350.Only the card and malaria tablets would cost around Rs.150, with the antibiotic it will cost a bit more. However, we have to inject most patients as they demand it. [Less qualified provider, Sundargarh]"

"When we realise that one of us needs money for medical purpose, we loan from our group (self-help group) at nominal interest with flexible repayment period. Like this we have supported many of us. [Female FGD participant, Mayurbhanj]"

On the other hand, LQPs have certain natural advantages because of their geographical proximity and flexibility in modes of payment, which can be paid in kind or in instalments. Visiting a far-off government health centre can be time consuming, expensive and inconvenient if regular transport facilities are not available. In contrast, LQPs would visit the household on receiving a phone call. There are community health workers in the villages or in the neighbourhoods providing care free of cost, but they hardly get recognized as they do not use RDT or ‘inject’ medicines.

"By realizing our financial condition, he (LQP) receives the payment when we can afford. This payment takes place within two to three days when he visits us for the injection. At times he allows us a month or two. We arrange money by borrowing from the neighbours or the moneylender at 5% interest rate. Some mortgage or sell their goats, bullocks and even land. [Male FGD participant, Sundargarh]"

"Here more people get treated in credit and repay the amount within two to three months. [Less qualified provider, Mayurbhanj]"

"You see…the ASHA in the village does not have card test (RDT) and injections. How can we expect quick cure if you don’t have these? [Male FGD participant, Mayurbhanj]"

The choice of providers is driven by faith and convenience (proximity, flexible payment modes, and perceived quick relief). Although most villages have a community health worker, the community does not have faith in them. The CHW does not have community’s acceptance for treatment of fever and malaria-like illnesses as there are frequent drug stock-outs.

"Whenever we go to them (CHW), they would tell that medicines are not there, so we do not go to them nowadays. [Male FGD participant, Sundargarh]"

## Discussion

Despite increasing investments in malaria control, access to prompt and effective treatment has remained a major challenge in most endemic settings [12]. The inability to consider local contexts, perceptions and cultural dynamics while designing policies for malaria control can lead to suboptimal community acceptance.

### Local illness concepts

This study found that the community had adopted the biomedical-equivalent term of malaria, known as *‘meleria’* to describe a broad range of illnesses. Studies from Tanzania and The Philippines also showed a similar phenomenon where local nomenclatures have evolved from the biomedical term [13,14]. This phenomenon may be due to their frequent exposure to the disease in the family and neighbourhood leading to regular interactions with the service providers. Further, awareness generation activities conducted in the community by the DoHFW and NGOs could be a contributor in this regard. The community’s ranking of malaria as the most common disease was in tandem with the actual prevalence of disease and service providers’ opinions.

Malaria was perceived primarily to be a water-borne disease with faeco-oral mode of transmission. Though the community recognized the role of mosquitoes in causing malaria, the perceived mechanism of disease transmission was often incorrect. Many stated multiple non-biomedical causes of disease transmission as reported from other endemic settings in Africa and Southeast Asia [15-21]. It is worth noting that lack of proper understanding of the causal link between the disease and vector leads to inadequate use of preventive methods. This was evident in this study as the participants reported the use of inappropriate personal and environmental hygiene measures to prevent malaria.

Though community knowledge of the causes of malaria was not fully accurate, the symptoms enumerated were very similar to the clinical presentations. Respondents could clearly differentiate fever due to malaria from other fevers and were aware of the complications of malaria if not treated on time. This could be due to the community members’ personal experiences of illness and the health awareness messages through community level health service providers. Women were found to be more aware of the symptoms than men, which could be explained by their role as the primary caregiver at home and close link with the female community health volunteers (ASHA).

### Prevention modalities

The use of prevention methods was determined by four factors; (1) perception of causes and disease transmission; (2) mosquito nuisance; (3) affordability and (4) climate. The reported practices on maintaining personal and environmental hygiene for malaria prevention were consistent with the local perception of causes and disease transmission. Examples from the African settings also demonstrate incorrect perceptions of disease transmission leading to inappropriate preventive behaviours without any change in malaria incidence [6,18].

Mosquitoes were perceived more as a nuisance than a vector that spreads malaria. Thus, the adoption of prevention methods was confined only to seasons with high vector densities as evidenced from endemic settings in Africa [22-26]. Almost all participants reported adopting some method of driving the mosquitoes away during the rainy season, when their population substantially increases. Fumigation of the house by burning dry leaves and wood in the evenings was the most prevalent prevention method. The protection offered by this kind of fumigation will be the least when most malaria-spreading mosquitoes (even though less in number) bite late in the night. This specific behaviour fails to recognize that malaria vectors can effectively transmit the disease even at low densities.

Affordability was a determinant of mosquito-net ownership, though the net was perceived to be an effective tool for protection from mosquito bites. Large family size and sleeping patterns would require a rural household to purchase multiple nets, which is beyond the financial capacity of many households. Considering the impoverished and vulnerable status of tribal communities, there is a clear ground for the state to provide them with either free or subsidized mosquito nets. Examples from Africa demonstrate improved health-seeking behaviour and health status after mass distribution of bed nets to vulnerable populations [27-30] and this constraint is expected to be somewhat alleviated under the new NVBDCP strategy that will distribute two LLINs free of cost to every household [8].

However, providing bed nets alone may not be sufficient given the sociocultural perceptions and behavioural patterns of the community. The use of bed nets was rather limited for malaria control as mosquito bites were not perceived to be the only cause of malaria. The alternative uses of bed nets were reported in this study; due to the intricacies of cultural and livelihood compulsions. This is also reported by studies from Solomon Islands and Kenya [22,31]. Learning lessons from earlier experiences, sustained behaviour-change communication (BCC) activities may be undertaken post-distribution to ensure the nets are being used appropriately. Inconsistent use of nets during the hot and humid nights due to physical discomfort has been reported from Africa and Asia [32-34]. Provision of bed nets with larger mesh size, which allow ventilation during summer nights, may be a potential solution to this problem prevalent in tropical and sub-tropical climates. There is an evidence of young children and mothers receiving priority use of bed nets as in other Afro-Asian settings [35,36], consistent with the public health messaging.

### Care seeking for febrile illnesses

**Figure 2:**
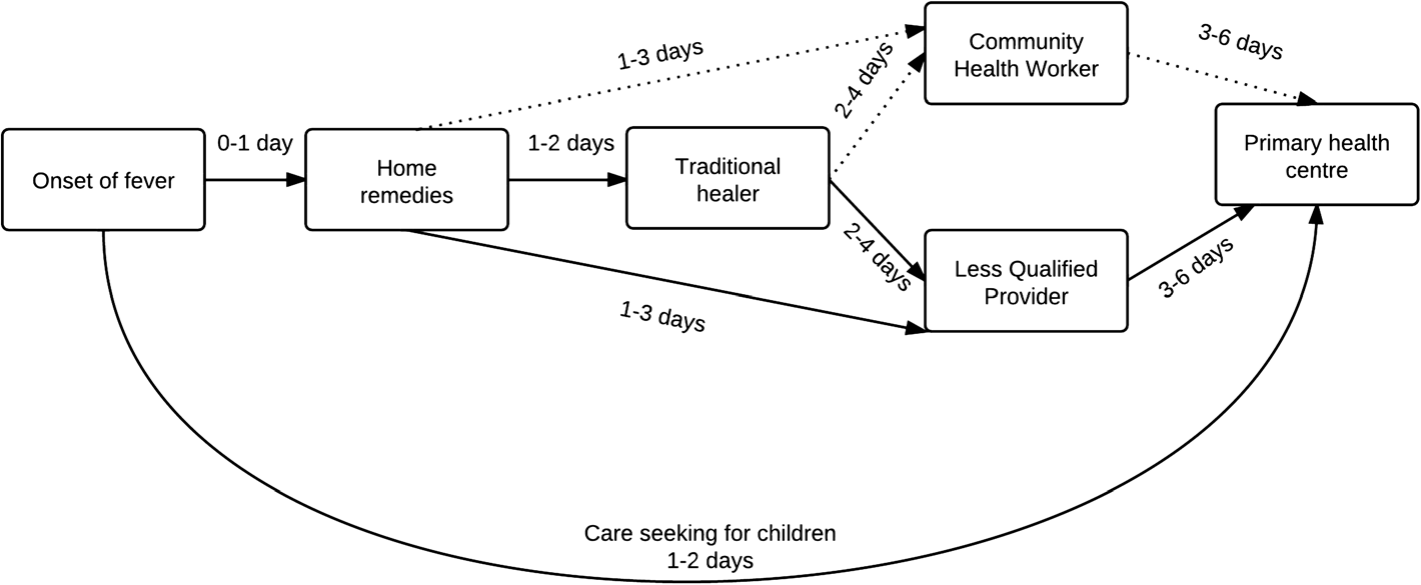
**Reported common pathways and duration to care seeking for febrile illness.** Notes: Number of days denotes duration of care seeking from the day of onset of symptoms; straight and curved arrows denote adult and child care seeking respectively; dotted arrows show obsolete pathways of care seeking after negative experiences with community health worker.

Care seeking for fever was found to be a complex interaction between sociocultural belief, risk perception, economic and livelihood factors. The pathways of care seeking for adult members consisted of multiple modalities as found in other endemic settings [37-39]. For most adults (Figure 2), it started typically with home remedies, followed by faith healing, community health worker or LQP, and only continued up to the primary health centre when complete cure was not yet achieved. The preference of home and traditional remedies could be explained by its low cost and easy availability as well as the community’s faith on traditional methods of healing.

Care from modern health care practitioners was only sought when it could not be managed by the local providers. However, the primary health centre was the first point of contact for sick infants and young children as the physician of the health centre was thought to possess the most necessary skills. Like most traditional societies, faith healers remained the first point of contact outside the household and public health system [15,40]. The public health system can leverage this community role of traditional healers to galvanize appropriate community behaviour. Possibly they can be included in the malaria control programme either as counsellors who direct patients to appropriate treatment providers or as drug distributors.

There were reported delays of more than 48 hours in care seeking for most uncomplicated episodes, which is a matter of concern for an area with high incidence of falciparum malaria. In a matter of few hours, falciparum malaria can progress towards severe and fatal complications [41]. At the village level, though the CHWs have been trained to dispense anti-malarials, there are frequent drug stock-outs due to inherent problems in supply-chain management. If a few febrile patients return empty handed from the CHW, it leads to negative publicity and others start to look for alternative service providers. Lack of faith in the CHW due to unavailability of drugs has been observed by earlier studies conducted in similar settings [21,42].

This study showed that cost of care for malaria in rural areas can be substantial. The households have to spend a quarter of their monthly income for a single episode of uncomplicated malaria. Though participants cited cost as a deterrent for acquiring nets, in fact they spend two to three times the price of a single net to treat one episode of malaria. This could be due to the desperation to treat malaria as early as possible so that livelihoods can be restored. Perceived ineffectiveness of nets in preventing malaria may also be a deterrent to net purchase and usage. On the other hand, higher prices charged to poor households by the LQPs through over-prescription could add to the financial burden of the household. This calls for alternative strategies for LQPs including the possible mainstreaming of LQPs into malaria control after adequate capacity development, as suggested by examples from Kenya and Nigeria [43,44].

Qualitative studies have their limitations in being less generalizable to larger contexts. Though limited in geographic and cultural scope, most of the findings in this study are similar to many endemic settings locally and globally. Adequate care has been undertaken to ensure representativeness of the study setting by including participants from a wide sociocultural, demographic and economic spectrum. Opinions from the perspectives of the service providers and community opinion leaders were collected and triangulated with that of the community members.

### Policy implications

Local community beliefs about disease transmission, availability and perceived quality of services are directly linked with health-seeking behaviour. For instance, provision of free bed nets might not induce adequate utilization if the majority of the population believes malaria is transmitted by contaminated water. Despite large investments in health infrastructure and human resources, if the programme does not take these beliefs into account during its planning and implementation, the change in health-seeking behaviour might not be adequate. India has reached a crucial juncture with revised strategies such as ACT and LLIN in its fight against malaria. In the context of introduction of more effective and expensive methods (eg, ACT and LLIN), it becomes imperative to ensure adequate and effective utilization. Evidence has shown that communities adopt practices if they have ownership of the intervention rather than imposing a ‘top-down’ approach [45-47]. The village health and sanitation committees (VHSC) under the framework of National Rural Health Mission and self-help groups in India are such forums for community participation in community health interventions. Adequate capacity development along with the provision of funds and technical supervision can enable committees to design, implement and monitor community-based malaria control interventions. For example, CBOs can support the distribution and subsequent monitoring of LLIN usage, sensitize the community about the availability of malaria diagnosis and treatment services with the ASHA, supervise indoor residual spray, undertake vector source reduction activities and track and monitor the trend of local fever and malaria cases. Simultaneously, the health system has to ensure regular availability of commodities so that the community does not lose faith in the ASHA and drift towards irrational and more expensive treatment methods. Implementation of the malaria control activities in integration with other health and disease control activities through the VHSC will help achieve the broader goal of primary health care.

## Abbreviations

ACT: Artemisinin-based combination therapy; ASHA: Accredited Social Health Activist; BCC: Behaviour change communication; CBO: Community based organizations; CHW: Community health worker; DoHFW: Department of Health and Family Welfare; FGD: Focus group discussion; KII: Key informant interview; LLIN: Long-lasting insecticide-treated bed nets; LQP: Less qualified provider; NVBDCP: National Vector Borne Diseases Control Programme; RDT: Rapid diagnostic test; VHSC: Village health and sanitation committee.

## Competing interests

The authors declare that they have no competing interests.

## Authors’ contributions

AD, JF, RD, MP, CM, and DS designed the study; AD, CM, DS collected and analysed the data. All authors read and approved the final draft.

## References

[B1] World Health Organization World malaria report 2008 Geneva: World Health Organization

[B2] World Health Organization World malaria report 2012 Geneva: World Health Organization

[B3] National vector borne disease control programme Malaria situation in India Government of India, Ministry of Health and Family Welfare http://nvbdcp.gov.in/Doc/mal-situation-Oct12.pdf [Accessed on 08 January 2013]

[B4] Kumar A Valecha N Jain T Dash AP Burden of malaria in India: retrospective and prospective view AmJTrop Med Hyg 2007 77 69 78 18165477

[B5] Dash AP Valecha N Anvikar AR Kumar A Malaria in India: challenges and opportunities J Biosci 2008 33 583 592 10.1007/s12038-008-0076-x 19208983

[B6] Ng’ang’a PN Jayasinghe G Kimani V Shililu J Kabutha C Kabuage L Githure J Mutero C Bed net use and associated factors in a rice farming community in Central Kenya Malar J 2009 8 64 10.1186/1475-2875-8-64 19371407 PMC2674467

[B7] Granado S Manderson L Obrist B Tanner M Appropriating “malaria”: local responses to malaria treatment and prevention in Abidjan, Cote d’Ivoire Med Anthropol 2011 30 102 121 10.1080/01459740.2010.488664 21218358

[B8] Government of India Operational Manual for Implementation of Malaria Programme 2009 New Delhi: Ministry of Health and Family Welfare

[B9] District profile, Mayurbhanj district Government of Odisha http://mayurbhanj.nic.in/ [Accessed on 10 March 2012]

[B10] District profile, Sundargarh district Government of Odisha http://sundergarh.nic.in/Introduction.htm [Accessed on 10 March 2012]

[B11] Hsieh HF Shannon SE Three approaches to qualitative content analysis Qual Health Res 2005 15 1277 1288 10.1177/1049732305276687 16204405

[B12] World Health Organization World malaria report 2011 Geneva: World Health Organization

[B13] Espino F Manderson L Acuin C Domingo F Ventura E Perceptions of malaria in a low endemic area in the Philippines: transmission and prevention of disease Acta Trop 1997 63 221 239 10.1016/S0001-706X(96)00623-7 9088436

[B14] Minja H Schellenberg JA Mukasa O Nathan R Abdulla S Mponda H Tanner M Lengeler C Obrist B Introducing insecticide-treated nets in the Kilombero Valley, Tanzania: the relevance of local knowledge and practice for an information, education and communication (IEC) campaign Trop Med Int Health 2001 6 614 623 10.1046/j.1365-3156.2001.00755.x 11555427

[B15] Vijayakumar KN Gunasekaran K Sahu SS Jambulingam P Knowledge, attitude and practice on malaria: a study in a tribal belt of Orissa state, India with reference to use of long lasting treated mosquito nets Acta Trop 2009 112 137 142 10.1016/j.actatropica.2009.07.011 19631184

[B16] Chibwana AI Mathanga DP Chinkhumba J Campbell CH Jr Socio-cultural predictors of health-seeking behaviour for febrile under-five children in Mwanza-Neno district, Malawialar J 2009 8 219 10.1186/1475-2875-8-219 PMC2763003 19778433

[B17] Howard N Shafi A Jones C Rowland M Malaria control under the Taliban regime: insecticide-treated net purchasing, coverage, and usage among men and women in eastern Afghanistan Malar J 2010 9 7 10.1186/1475-2875-9-7 20053281 PMC2817706

[B18] Dye TD Apondi R Lugada ES Kahn JG Smith J Othoro C “Before we used to get sick all the time”: perceptions of malaria and use of long-lasting insecticide-treated bed nets (LLINs) in a rural Kenyan community Malar J 2010 9 345 10.1186/1475-2875-9-345 21118550 PMC3225033

[B19] Adongo PB Kirkwood B Kendall C How local community knowledge about malaria affects insecticide-treated net use in northern Ghana Trop Med Int Health 2005 10 366 378 10.1111/j.1365-3156.2005.01361.x 15807801

[B20] Das A Ravindran TS Factors affecting treatment-seeking for febrile illness in a malaria endemic block in Boudh district, Orissa, India: policy implications for malaria control Malaria J 2010 9 377 10.1186/1475-2875-9-377 PMC3224374 21192825

[B21] Das A Sundari Ravindran TK Community knowledge on malaria among febrile patients in an endemic district of Orissa, India J Vector Borne Dis 2011 48 46 51 21406737

[B22] Atkinson JA Bobogare A Fitzgerald L Boaz L Appleyard B Toaliu H Vallely A A qualitative study on the acceptability and preference of three types of long-lasting insecticide-treated bed nets in Solomon Islands: implications for malaria elimination Malar J 2009 8 119 10.1186/1475-2875-8-119 19497127 PMC2699345

[B23] Atkinson JA Fitzgerald L Toaliu H Taleo G Tynan A Whittaker M Riley I Vallely A Community participation for malaria elimination in Tafea Province, Vanuatu: Part I. Maintaining motivation for prevention practices in the context of disappearing disease Malar J 2010 9 93 10.1186/1475-2875-9-93 20380748 PMC2873527

[B24] Sood RD Mittal PK Kapoor N Razdan RK Dua VK Dash AP Community awareness, perceptions, acceptability and preferences for using LLIN against malaria in villages of Uttar Pradesh, India J Vector Borne Dis 2010 47 243 248 21178218

[B25] Snehalatha KS Ramaiah KD Vijay Kumar KN Das PK The mosquito problem and type and costs of personal protection measures used in rural and urban communities in Pondicherry region, South India Acta Trop 2003 88 3 9 10.1016/S0001-706X(03)00155-4 12943970

[B26] Peeters Grietens K Xuan XN Van Bortel W Duc TN Ribera JM Ba Nhat T Van KP Le Xuan H D’Alessandro U Erhart A Low perception of malaria risk among the Ra-glai ethnic minority in south-central Vietnam: implications for forest malaria control Malar J 2010 9 23 10.1186/1475-2875-9-23 20089152 PMC2823606

[B27] Terlouw DJ Morgah K Wolkon A Dare A Dorkenoo A Eliades MJ Vanden Eng J Sodahlon YK ter Kuile FO Hawley WA Impact of mass distribution of free long-lasting insecticidal nets on childhood malaria morbidity: the Togo National Integrated Child Health Campaign Malar J 2010 9 199 10.1186/1475-2875-9-199 20624305 PMC2914062

[B28] Gerstl S Dunkley S Mukhtar A Maes P De Smet M Baker S Maikere J Long-lasting insecticide-treated net usage in eastern Sierra Leone - the success of free distribution Trop Med Int Health 2010 15 480 488 20149163 10.1111/j.1365-3156.2010.02478.x

[B29] de Oliveira Macedo A Wolkon A Krishnamurthy R Erskine M Crenshaw DP Roberts J Saute F Ownership and usage of insecticide-treated bed nets after free distribution via a voucher system in two provinces of Mozambique Malar J 2010 9 222 10.1186/1475-2875-9-222 20684764 PMC2925365

[B30] Beer N Ali AS de Savigny D Al-Mafazy AW Ramsan M Abass AK Omari RS Bjorkman A Kallander K System effectiveness of a targeted free mass distribution of long lasting insecticidal nets in Zanzibar, Tanzania Malar J 2010 9 173 10.1186/1475-2875-9-173 20565860 PMC2911471

[B31] Minakawa N Dida GO Sonye GO Futami K Kaneko S Unforeseen misuses of bed nets in fishing villages along Lake Victoria Malar J 2008 7 165 10.1186/1475-2875-7-165 18752662 PMC2532690

[B32] Ordinioha B The use of insecticide-treated bed net in a semi-urban community in south-south, Nigeria Niger J Med 2007 16 223 226 17937157

[B33] Korenromp EL Miller J Cibulskis RE Kabir Cham M Alnwick D Dye C Monitoring mosquito net coverage for malaria control in Africa: possession vs. use by children under 5 years Trop Med Int Health 2003 8 693 703 10.1046/j.1365-3156.2003.01084.x 12869090

[B34] Yohannes K Dulhunty JM Kourleoutov C Manuopangai VT Polyn MK Parks WJ Williams GM Bryan JH Malaria control in central Malaita, Solomon Islands. 1. The use of insecticide-impregnated bed nets Acta Trop 2000 75 173 183 10.1016/S0001-706X(00)00055-3 10708657

[B35] Tsuang A Lines J Hanson K Which family members use the best nets? An analysis of the condition of mosquito nets and their distribution within households in Tanzania Malar J 2010 9 211 10.1186/1475-2875-9-211 20663143 PMC2918626

[B36] Githinji S Herbst S Kistemann T Noor AM Mosquito nets in a rural area of Western Kenya: ownership, use and quality Malar J 2010 9 250 10.1186/1475-2875-9-250 20813034 PMC2939624

[B37] Munguti KJ Community perceptions and treatment seeking for malaria in Baringo district, Kenya: implications for disease control East Afr Med J 1998 75 687 691 10065206

[B38] Jombo GTA Mbaawuaga EM Denen AP Dauda AM Eyong KI Akosu JT Etukumana EA Utilization of traditional healers for treatment of malaria among female residents in Makurdi city and its environs Asian Pac J Trop Med 2010 3 563 566 10.1016/S1995-7645(10)60136-8

[B39] Espino F Manderson L Treatment seeking for malaria in Morong, Bataan, the Philippines Soc Sci Med 2000 50 1309 1316 10.1016/S0277-9536(99)00379-2 10728850

[B40] Foster D Vilendrer S Two treatments, one disease: childhood malaria management in Tanga, Tanzania Malar J 2009 8 240 10.1186/1475-2875-8-240 19860900 PMC2779815

[B41] Greenwood BM Bradley AK Greenwood AM Byass P Jammeh K Marsh K Tulloch S Oldfield FS Hayes R Mortality and morbidity from malaria among children in a rural area of The Gambia, West Africa Trans R Soc Trop Med Hyg 1987 81 478 486 10.1016/0035-9203(87)90170-2 3318021

[B42] Das LK Jambulingam P Sadanandane C Impact of community-based presumptive chloroquine treatment of fever cases on malaria morbidity and mortality in a tribal area in Orissa State, India Malar J 2008 7 75 10.1186/1475-2875-7-75 18457582 PMC2390570

[B43] Abuya T Fegan G Rowa Y Karisa B Ochola S Mutemi W Marsh V Impact of ministry of health interventions on private medicine retailer knowledge and practices on anti-malarial treatment in Kenya AmJTrop Med Hyg 2009 80 905 913 19478247

[B44] Okeke TA Uzochukwu BS Improving childhood malaria treatment and referral practices by training patent medicine vendors in rural south-east Nigeria Malar J 2009 8 260 10.1186/1475-2875-8-260 19930561 PMC2784476

[B45] Ghebreyesus TA Alemayehu T Bosman A Witten KH Teklehaimanot A Community participation in malaria control in Tigray region Ethiopia Acta Trop 1996 61 145 156 10.1016/0001-706X(95)00107-P 8740892

[B46] le Hung Q Vries PJ Giao PT Nam NV Binh TQ Chong MT Quoc NT Thanh TN Hung LN Kager PA Control of malaria: a successful experience from Viet Nam Bull World Health Organ 2002 80 660 666 12219158 PMC2567582

[B47] Freeman T Community-based malaria control in Zimbabwe Bull World Health Organ 1999 77 295 296 10212530 PMC2557628

